# Pharmaceutical company payments to authors of the Japanese guidelines for the management of hypertension

**DOI:** 10.1097/MD.0000000000024816

**Published:** 2021-03-26

**Authors:** Yuki Senoo, Hiroaki Saito, Akihiko Ozaki, Toyoaki Sawano, Yuki Shimada, Kana Yamamoto, Yosuke Suzuki, Tetsuya Tanimoto

**Affiliations:** a Medical Governance Research Institute, Shinagawa, Tokyo; b Department of Gastroenterology, Sendai Kousei Hospital, Sendai, Miyagi; c Department of Breast Surgery, Jyoban Hospital of Tokiwa Foundation, Iwaki, Fukushima; d Department of Surgery, Sendai City Medical Center, Sendai, Miyagi; e Department of Neurosurgery, Minamisoma Municipal General Hospital, Minamisoma, Fukushima; f Department of Obstetrics and Gynecology, Tone Chuo Hospital, Gunma, Japan.

**Keywords:** financial conflicts of interest, hypertension guidelines, Japan, pharmaceutical industry

## Abstract

Antihypertensive drugs have been of significant interest to the pharmaceutical industry due to increasing sales opportunities in a global market. The financial relationships between pharmaceutical companies and the Japanese Society of Hypertension (JSH) have a possible influence on clinical practices in Japan. This study examined the distribution of pharmaceutical payments made to the authors of the revised Guidelines for the Management of Hypertension (JSH2019) and the transparency of the Conflict of Interest disclosure that each author made.

We retrospectively obtained publicly available data regarding payments made by Japanese pharmaceutical companies to all authors of the JSH2019 in 2016. We also collected data on individual financial disclosure of JSH2019 authors to investigate whether their self-reported financial relationship with companies were compliant to the financial disclosure policy of JSH2019.

The total and mean payment values reported by pharmaceutical companies were $4,246,436 and $21,447, respectively. Of the 198 authors, 171 (86.4%) authors received at least 1 payment. Of 74 authors required to disclose their conflict of interest (COI) the authors, one-third failed to follow the COI policy covering the clinical guidelines.

Major pharmaceutical companies selling antihypertensive drug products in the Japanese market had a significant financial connection with the JSH2019 authors. Financial relationships between pharmaceutical companies and authors or Japanese medical societies are raising significant concerns about the credibility of clinical guidelines and the potentially biases and undue influences that they may cause, especially with respect to adverse prescription patterns.

## 1 Introduction

Antihypertensive drugs are attractive targets for the pharmaceutical industry due to their high income-generating potential. In April 2019, the Japanese Society of Hypertension (JSH) published a set of revised Guidelines for the Management of Hypertension (JSH2019).^[1]^ The new Guidelines recommended a lower target blood pressure level of <130/80 mm Hg, mirroring the 2017 American College of Cardiology (ACC)/American Heart Association (AHA) guidelines. However, the scope of antihypertensive treatment in the Japanese population has not been fully investigated,^[2]^ which raises concerns about possible unwarranted pharmaceutical industry influences.

Today, the first-line antihypertensive drugs used in practice comprise 4 main classes: angiotensin-converting enzyme (ACE) inhibitors, angiotensin II receptor blockers (ARBs), thiazide diuretics, and calcium channel blockers (CCBs). Both the ACC (AHA2017 guidelines) and JSH2019, mention that there are no significant differences in the effect and safety of antihypertensive drugs in each class in lowering blood pressure among 30% to 50% of patients.^[2,3]^ Therefore, the general principal of hypertension management is to choose any or the most appropriate or available drug, unless the patient has a specific clinical reason otherwise, such as contraindication or pregnancy. Furthermore, observations suggest that reduction in cardiovascular risk is also independent of the drug choice, being due simply to the amount of blood pressure reduction.^[4,5]^ In response to this, pharmaceutical companies manufacture a variety of antihypertensive drugs meaning there is a wide variety of quite similar drugs competing in the market. In such a competitive market, the marketing and promotional activities of pharmaceutical companies are almost certain to be tightly focused on those individuals who have a major influence on prescription policies and behavior. Considering the extensive recent clinical trial misconduct of Valsartan, an ARB manufactured by Novartis, involving JSH members^[6]^ and their still unchanged financial tie with pharmaceutical companies after the scandal,^[7]^ the authors of clinical practice guidelines (CPGs) should be completely transparent about any potential conflicts of interest (COIs), especially with respect to links with the pharmaceutical industry. We consequently investigated the extent and characteristics of financial COIs declared by the JSH2019 authors.

## 2 Methods

The 78 pharmaceutical companies belonging to the Japanese Pharmaceutical Manufacturers Association (JPMA) disclose data of payments that they make to all Japanese healthcare professionals as required under the voluntary Transparency Guideline for the Relation between Corporate Activities and Medical Institutions, which governs all JPMA member companies. We obtained data on payments made by JPMA member companies for lecturing, writing, and consulting to all JSH authors in 2016 using methods as previously described.^[8]^ These payments are usually counted as incomes of the authors, not as research funding or other donations. We also collected relevant data, including names, affiliations, positions of the authors from their affiliations’ and other websites. Japanese yen were converted to United States of America dollars using the 2016 average monthly exchange rate of 108.8 yen per 1 US dollar.

We evaluated the accuracy of the financial COIs reported by the authors, by comparing them with the payments disclosed by the pharmaceutical companies; the JSH2019 has a COI disclosure section requiring named authors provide mandatory details of any monies received, in excess of ¥500,000 ($4596), from any pharmaceutical company for lecturing or writing work. Payments for consulting were excluded in this analysis as they were not covered in the JSH2019 COI disclosure requirements. Institutional ethical committee approval was obtained from the Committee on the Medical Governance Research Institute, Minato-ku, Tokyo, Japan. Informed consent from the JSH2019 authors was not obtained as the payment data included in this study was collected from the publicly available data disclosed on the official homepage of each pharmaceutical company.

## 3 Results

The JSH2019 had 198 authors: 146 (73.7%) were employees at universities or university hospitals and 92 (46.5%) were university professors. Payments totalled $4,246,436 (see summary in Table 1). The median and mean payment values for each author were $6316 (interquartile range: $1210–$ 32,603) and $21,447 (standard deviation: $32,722), respectively. Among 171 (86.4%) authors who received at least 1 payment, 103 (60.2%) and 82 (48.0%) received $5000 and $10,000 or above, respectively. The top 20 (10%) authors received $2,030,448 (47.8%) of the total payments, of whom 18 (90%) were professors. Daiichi Sankyo Company Ltd., Boehringer Ingelheim Japan, and Takeda Pharmaceutical Company Ltd. were the companies reporting the largest payments. Of the 74 authors required to disclose their financial COIs under the JSH2019 criteria, 27 (36.5%) failed to do so.

**Table 1:** Characteristics of clinical guideline authors and pharmaceutical company payments.

Variables	Authors (n = 198)
Affiliations	
Universities	107 (54.0%)
University hospitals	39 (19.7%)
Other types of hospitals	32 (16.2%)
Research institutes	6 (3.0%)
Clinics	6 (3.0%)
Others	8 (4.0%)
University professors	
Yes	92 (46.5%)
No	106 (53.5%)
Authors with payments	
Median (interquartile range)	$6316 ($1210–$32,603)
Mean (standard deviation)	$21,447 ($32,722)
Any	171 (86.4%)
≧$5000	103 (52.0%)
≧$10,000	82 (41.5%)
Payments from top 3 companies	
Daiichi-Sankyo Company, Limited	$560,481
Nippon Boehringer Ingelheim Co., Ltd.	$471,277
Takeda Pharmaceutical Company Ltd.	$393,348

## 4 Discussion

Our study confirmed that JSH2019 authors received sizable payments; the mean payment values for each author accounts for approximately 18% to 21% of the average annual salary of Japanese physicians,^[9]^ from companies whose commercial products were directly related to guideline topics. Furthermore, in the previous studies, similar financial ties were also observed between pharmaceutical companies and Japanese CPG authors in other following fields: dermatology,^[10]^ dementia,^[8]^ MRSA,^[11]^ Hepatitis C treatment,^[12]^ hematology,^[13]^ urology,^[14]^ and oncology.^[15]^ The mean payment values to JSH2019 authors fall in the range of those received by CPG authors covering other clinical field, from $10,565 to $66,979.^[8,10–15]^ Reflecting on this point, our study finding suggests that JSH2019 authors also had a major financial COIs which is an extension of deeply rooted issue surrounding medical field remained underreported.

Of the top 5 companies registering the largest payments to JSH2019 authors, 3 sold antihypertensive drugs with a reported sales income of more than ¥5 billion ($46 million) in 2018.^[16]^ We suspect that due to the highly competitive and lucrative hypertension drug market and a continually increasing number of patients, the marketing strategy of companies with antihypertensive products for sale may have preferentially targeted the most influential individuals, indicated by the fact that the majority of the 20 JSH2019 authors with the highest payments were university professors. Furthermore, in both studies, it was revealed that all of the top 5 largest paying companies manufactured the products relevant to the topic of each guideline.

Notably, a third of JSH2019 authors clearly failed to comply with COI regulations, despite serious misconduct in Japan that led to the 2018 rigid enforcement of the Clinical Trials Act by Japan's Ministry of Health, Labour and Welfare. Comparably, the previous investigation regarding pharmaceutical payment received by Japanese CPG authors also documented that under-reported and imprecise FCOI were found in profound number of authors.^[8,10–15]^ This damages the credibility and trustworthiness of the JSH2019, especially as the update is advantageous for the pharmaceutical industry. Clearly, it is imperative that author COI disclosure policies need to be both clarified and rigorously enforced. Further, all authors need to comply and guideline publishers have to take responsibility to ensure that all requirements have been met in full and that all necessary details are published accurately.

As explained above, financial ties between the CPG authors and pharmaceutical companies are prevalent and still ongoing issue surrounding clinical practice in Japan.^[8,10–15]^ As a countermeasure, the recruitment of CPG authors should be revised. It is true that a balanced compilation of CPG authors requires healthcare professionals, namely physicians, with abundant clinical experience and knowledge about the latest evidence in any specific medical field.^[17]^ However, it is to be noted that Japanese physicians mainly receive payments from pharmaceutical companies by serving as lecturers and often leave their own hospitals and travel throughout the country. In this regard, it is questionable how familiar these physicians are with current frontline clinical practice.

Limitations in our study include potential payment underestimates due to lack of information regarding research funding, donations, travel fees, meals, and other gifts, while payments from the medical device industry could not be included. Further, our manual handling of large volume payments data may have produced some errors in estimation of individual payment values.

## 5 Conclusion

In conclusion, we demonstrated significant financial relationships between pharmaceutical industry and the authors of JSH2019 even after the unprecedented misconduct related to antihypertensive drugs. Given the long-standing ties between the pharmaceutical industry and Japanese medical societies, significant concerns exist about the ethical and potentially corrupt nature of these relationships, which could cause biased and potentially harmful prescription patterns, as well as increased health care costs that could threaten the sustainability of Japan's globally-acclaimed universal health coverage and world-leading health care system.

## Author contributions

**Conceptualization:** Akihiko Ozaki, Tetsuya Tanimoto.

**Data curation:** Hiroaki Saito.

**Formal analysis:** Yuki Senoo, Hiroaki Saito, Toyoaki Sawano.

**Investigation:** Hiroaki Saito, Akihiko Ozaki, Toyoaki Sawano.

**Supervision:** Tetsuya Tanimoto.

**Visualization:** Yousuke Suzuki, Tetsuya Tanimoto.

**Writing – original draft:** Yuki Senoo, Akihiko Ozaki.

**Writing – review & editing:** Yuki Senoo, Hiroaki Saito, Akihiko Ozaki, Toyoaki Sawano, Yuki Shimada, Kana Yamamoto, Yousuke Suzuki, Tetsuya Tanimoto.

## References

[1] Umemura S Arima H Arima S. The Japanese Society of Hypertension Guidelines for the Management of Hypertension (JSH 2019). Hypertens Res 2019;42:1235–481. 31375757 10.1038/s41440-019-0284-9

[2] Whelton PK Carey RM Aronow WS. 2017 ACC/AHA/AAPA/ABC/ACPM/AGS/APhA/ASH/ASPC/NMA/PCNA guideline for the prevention, detection, evaluation, and management of high blood pressure in adults: executive summary: a report of the American College of Cardiology/American Heart Association Task Force on Clinical Practice Guidelines. Hypertension 2018;71:1269–324. 29133354 10.1161/HYP.0000000000000066

[3] Hirawa N Umemura S Ito S. Viewpoint on guidelines for treatment of hypertension in Japan. Circ Res 2019;124:981–3. 30920921 10.1161/CIRCRESAHA.119.314991

[4] Turnbull F Neal B Ninomiya T. Effects of different regimens to lower blood pressure on major cardiovascular events in older and younger adults: meta-analysis of randomised trials. BMJ 2008;336:1121–3. 18480116 10.1136/bmj.39548.738368.BE PMC2386598

[5] Ettehad D Emdin CA Kiran A. Blood pressure lowering for prevention of cardiovascular disease and death: a systematic review and meta-analysis. Lancet 2016;387:957–67. 26724178 10.1016/S0140-6736(15)01225-8

[6] Tanimoto T Kami M Shibuya K. Japan to learn from biomedical cases. Nature 2014;512:371–1371. 10.1038/512371d 25164738

[7] Sawano T Ozaki A Saito H. Payments from pharmaceutical companies to authors involved in the Valsartan Scandal in Japan. JAMA Netw Open 2019;2:e193817–193817. 31099864 10.1001/jamanetworkopen.2019.3817 PMC6537813

[8] Shimada Y Ozaki A Saito H. Pharmaceutical company payments to the authors of the Japanese dementia clinical practice guidelines in 2016. Alzheimers Dement (N Y) 2019;5:228–30. 31297436 10.1016/j.trci.2019.05.003 PMC6597934

[9] Ministry of Health, Labour and Welfare. Summary report of the Basic Survey on Wage Structure. February 22, 2017. Available at: https://www.e-stat.go.jp/stat-search/files?page=1&layout=datalist&toukei=00450091&tstat=000001011429&cycle=0&tclass1=000001074669&tclass2=000001074675&tclass3=000001074676 [accessed 30 September, 2020].

[10] Murayama A Ozaki A Saito H. Pharmaceutical company payments to dermatology Clinical Practice Guideline authors in Japan. PLoS One 2020;15:e0239610. 33048952 10.1371/journal.pone.0239610 PMC7553305

[11] Saito H Tani Y Ozaki A. Financial ties between authors of the clinical practice guidelines and pharmaceutical companies: an example from Japan. Clin Microbiol Infect 2019;25:1304–6. 31401175 10.1016/j.cmi.2019.07.025

[12] Kida F Murayama A Saito H. Pharmaceutical company payments to authors of the Japanese Clinical Practice Guidelines for Hepatitis C treatment [published online ahead of print, 2020 Dec 11]. Liver Int 2020;doi: 10.1111/liv.14761. 10.1111/liv.14761 33306236

[13] Harada K Ozaki A Saito H. Financial payments made by pharmaceutical companies to the authors of Japanese hematology clinical practice guidelines between 2016 and 2017 [published online ahead of print, 2020 Dec 17]. Health Policy 2020;doi: 10.1016/j.healthpol.2020.12.005. 10.1016/j.healthpol.2020.12.005 33386174

[14] Yamamoto K Murayama A Ozaki A. Financial conflicts of interest between pharmaceutical companies and the authors of urology clinical practice guidelines in Japan [published online ahead of print, 2020 Nov 5]. Int Urogynecol J 2020;doi: 10.1007/s00192-020-04547-3. 10.1007/s00192-020-04547-3 33151353

[15] Saito H Ozaki A Sawano T. Evaluation of pharmaceutical company payments and conflict of interest disclosures among oncology clinical practice guideline authors in Japan. JAMA Netw Open 2019;2:e192834–192834. 31026027 10.1001/jamanetworkopen.2019.2834 PMC6487566

[16] Insights4 Pharmaceutical. FY2018 Domestic Pharmaceutical Sales Ranking. June 30, 2019. Available at: http://pharma.insights4.jp/archives/14987 [accessed May 16, 2020].

[17] Scott IA Guyatt GH. Clinical practice guidelines: the need for greater transparency in formulating recommendations. Med J Aust 2011;195:29–33. 21728938 10.5694/j.1326-5377.2011.tb03184.x

